# Using electronic patient reported outcomes (ePROs) to improve supportive care in ambulatory oncology care: a CFIR-guided pre-implementation formative evaluation

**DOI:** 10.1186/s41687-026-01144-8

**Published:** 2026-07-21

**Authors:** Eden R. Brauer, Roshan Bastani, Eric Cheng, Stephanie Lazaro, Deborah J. Wong, Maie A. St. John, Patricia A. Ganz

**Affiliations:** 1 University of California, Los Angeles (UCLA), Los Angeles, USA; 2 UCLA Health, Los Angeles, USA

**Keywords:** Electronic patient-reported outcomes, Implementation science, CFIR, Head and neck cancer, Supportive care, Formative evaluation, Clinical decision support

## Abstract

**Background:**

Systematic collection of patient-reported outcomes via electronic health records can improve symptom assessment and supportive care delivery in oncology, yet implementation is often hindered by inadequate attention to end-user needs and organizational context. Guided by an implementation science framework, we conducted a formative evaluation to inform design and implementation planning of an electronic patient-reported outcome supportive care screening system in ambulatory oncology care, using head and neck cancer as a use case.

**Methods:**

A prospective mixed-methods observational study was conducted using semi-structured interviews as part of a pre-implementation formative evaluation. Eligible participants were target users of the proposed supportive care screening system, including (1) clinical staff and (2) patients with head and neck cancer from a single health care system. Using the Consolidated Framework for Implementation Research as a guiding framework, we elicited user feedback through interview and prototype engagement. Quantitative measures included patient symptom burden and validated pre-implementation measures of acceptability, appropriateness, feasibility, and usability. For data analyses, we used directed content analysis and matrix analysis for qualitative data, and descriptive and comparative statistics for quantitative assessments.

**Results:**

Forty-six target users (20 clinical staff, 26 patients) participated. Both groups expressed strong tension for change, recognizing many inadequacies of the current approach to symptom assessment. Clinical staff described current practices as “haphazard,” while patients recounted missed opportunities for support and preventable harm. Electronic health record integration enhanced perceived compatibility, though time constraints and alert fatigue emerged as potential barriers. Clinical staff emphasized the need for clear symptom thresholds and actionable referral criteria; patients emphasized that their sustained engagement depended on evidence of clinicians using the data in their care. Pre-implementation scores indicated strong readiness across both groups: acceptability (clinical staff 4.47/5, patients 4.21/5), appropriateness (4.46/5, 4.24/5), feasibility (4.41/5, 4.35/5), and usability (82.2/100, 85.6/100). Findings informed key design priorities and implementation strategies.

**Conclusions:**

Systematic pre-implementation formative evaluation revealed important barriers, facilitators, and contextual insights that shaped both intervention design and implementation approach. High scores on leading indicators suggest strong organizational readiness and perceived value. This user-informed development process provides a replicable model for optimizing electronic patient-reported outcome systems before deployment.

**Supplementary Information:**

The online version contains supplementary material available at https://doi.org/10.1186/s41687-026-01144-8.

## Introduction

Supportive care, which encompasses symptom management, as well as psychosocial, rehabilitative, survivorship, and palliative services, is fundamental to high-quality cancer care [1]. At its core, supportive care addresses physical, emotional, and social needs while enhancing patient-centered communication [2, 3]. With an estimated 18.6 million cancer survivors in the United States (U.S.) and projected growth to 22 million by 2035 [4], leading organizations have called for proactive integration of supportive care across the cancer care continuum, from diagnosis through survivorship [1, 2]. Despite strong evidence demonstrating that supportive care interventions improve symptom control, quality of life, and survival [5], integration into routine practice remains challenging, and many supportive care services do not reach patients who could benefit most [6].

This delivery gap stems primarily from two interrelated challenges: (1) effective identification of supportive care needs of patients during time-constrained encounters, and (2) optimal clinical response to identified needs in fragmented care settings. Without standardized assessment, clinicians underestimate or overlook up to 50% of patients’ symptoms [7–12]. Even when needs are identified, referral-based services remain underutilized, with connections to supportive care often dependent on individual provider knowledge rather than clear criteria or care pathways [13, 14].

Patient-reported outcomes (PROs) offer a promising solution to the first challenge. Considered the gold standard for symptom assessment, PROs provide a pragmatic strategy to capture symptoms and supportive care needs from the patient’s perspective in routine care [15, 16]. A recent meta-analysis demonstrates that PRO integration improves survival and quality of life through earlier need identification and timely response [5]. Based on these findings, leading professional entities now recognize electronic PRO (ePRO) symptom monitoring as a “digital therapeutic intervention” and have identified routine PRO collection in health records as a top priority for advancing timely, individualized supportive care for all people affected by cancer [2, 15].

However, ensuring appropriate clinical response to ePRO data requires mechanisms to translate identified needs into clinical action. While PRO monitoring alone has been shown to yield benefits, multicomponent systems incorporating structured clinical guidance consistently achieve superior clinical outcomes [17, 18]. Despite growing interest in ePROs, examples of real-world implementation in oncology care remain limited, and fewer still include mechanisms for structured clinical response linking ePROs to supportive care services [13, 15, 19].

Systems that integrate ePRO data with clinical decision support (ePRO + CDS) to guide supportive care delivery have emerged as a strategy to bridge this gap. Effective ePRO + CDS systems must also balance standardization for scalability with customization to address disease-specific concerns. In addition, many ePRO initiatives fail to achieve sustained adoption due to inadequate attention to organizational context, workflow integration, and end-user needs during system design [6].

To address these challenges, implementation science frameworks, such as the Consolidated Framework for Implementation Research (CFIR) [20, 21], offer systematic methods for formative evaluation [6, 22]. When applied during pre-implementation phases, framework-guided formative evaluation can inform both intervention development and implementation strategy to optimize conditions for successful adoption and sustainability [23].

Applying this approach, we selected head and neck cancer (HNC) as an initial use case to develop and test an ePRO + CDS system. Patients with HNC have complex, multidimensional supportive care needs affecting speech, swallowing, nutrition, pain, and psychosocial functioning [24, 25]. This complexity allows testing of whether a modular ePRO + CDS system—pairing core symptom assessment with disease-specific screening and tailored clinical decision support—can be successfully implemented in routine care while remaining scalable to other cancer populations [24–28].

This paper describes the pre-implementation formative evaluation conducted to inform design and implementation of a modular ePRO + CDS system in ambulatory oncology, using HNC as the initial use case. Guided by the CFIR, we conducted semi-structured interviews with target users, including both clinical staff and patients with HNC, and assessed pre-implementation indicators of success to systematically identify design requirements and implementation strategies prior to deployment.

## Methods

### Study design

We conducted a pre-implementation formative evaluation using mixed methods to inform design and strategic planning for an ePRO + CDS system to promote supportive care in ambulatory oncology. Formative evaluation aims to optimize implementation success by systematically assessing barriers and facilitators, feasibility, and target users’ perceptions of value [23].

The formative evaluation was guided by the CFIR version 2.0, which provides a comprehensive, evidence-based taxonomy of implementation determinants across five domains: Innovation, Outer Setting, Inner Setting, Individuals, and Implementation Process [21]. Given the pre-implementation timing, we focused the assessment on three domains most relevant to intervention development and preparation: Innovation (characteristics of the proposed ePRO system), Inner Setting (clinical context in which the tool will be implemented), and Individuals (characteristics, perspectives, and needs of target users) [23].

This study was designed and reported according to the Consolidated Criteria for Reporting Qualitative Research (COREQ) [29], with selected elements appropriate for pre-implementation from the Standards for Reporting Implementation Studies (StaRI) [30] (Supplementary Tables S1 and S2). The study received approval from the Institutional Review Board (IRB #20-1323).

### Setting and context

The study was conducted at a large academic medical center with an NCI-designated comprehensive cancer center in the Western U.S. We selected HNC for developing and piloting the ePRO + CDS approach given the complex supportive care needs, significant functional and psychosocial impairment, and intensified toxicity burden from multimodal treatments. Patients with HNC at this institution receive multidisciplinary care from surgical oncology, radiation oncology, and medical oncology, with access to supportive care services including speech-language pathology, nutrition, social work, psycho-oncology, rehabilitation, pain management, and palliative care. Prior to this study, no standard processes for symptom assessment or supportive care referrals were in place. A central consideration in this formative evaluation was patient portal access. At study onset, institutional data indicated that patient portal registration in the target clinics exceeded 90%, supporting the feasibility of a portal-delivered ePRO approach in this setting.

### Participants and recruitment

To inform development and implementation planning of the ePRO + CDS system, we recruited two groups of target users: (1) clinical staff involved in HNC care delivery, and (2) patients with HNC receiving care at the study institution. The clinical staff group included: clinicians (physicians, nurses) from oncology subspecialties (medical oncology, radiation oncology, or head and neck surgery); clinic staff and administrators involved in HNC care; and supportive care specialists (social work, nutrition, speech-language pathology, physical therapy, pain management, palliative care, psycho-oncology). Clinical staff were recruited via email invitation using purposeful sampling to identify information-rich cases and ensure diversity of perspectives [31].

Patients were identified through the institutional tumor registry based on the following eligibility criteria: HNC diagnosis within the past 2 years; currently receiving or completed HNC treatment; English-speaking; and ability to provide informed consent. Eligible patients were invited to participate by mail in randomly selected batches. Enrollment was based on response, resulting in a convenience sample. To incorporate a range of patient perspectives, we sequenced patient interviews as data collection progressed based on available registry characteristics (age, tumor site, and time since diagnosis).

Sample size was determined based on pragmatic considerations and recommendations for qualitative studies using framework-guided analysis in implementation science research [32]. Consistent with formative evaluation approaches, sampling continued until sufficient information was obtained across CFIR constructs to guide design decisions, rather than pursuing theoretical saturation [31].

### Prototype development

**Fig. 1:**
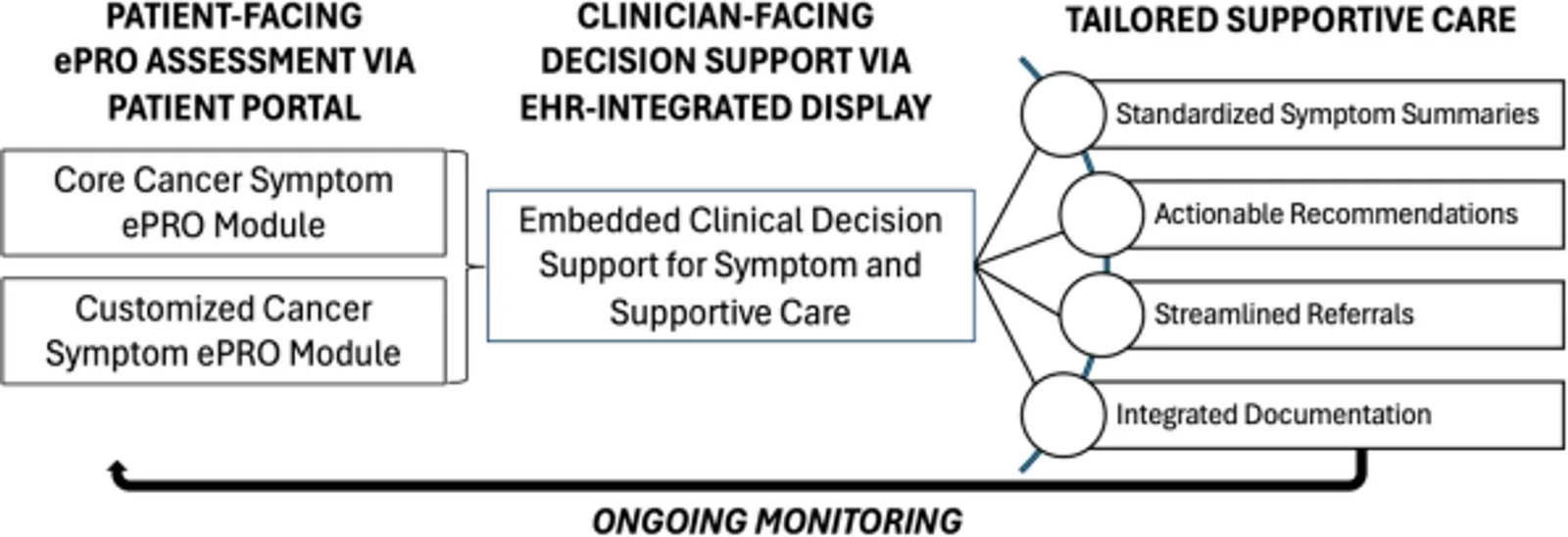
Modular electronic patient-reported outcome plus clinical decision support (ePRO + CDS) symptom screening framework. This figure illustrates the conceptual architecture and workflow of the proposed ePRO + CDS system. The patient-facing component, delivered via the electronic health record (EHR) patient portal, includes a core cancer symptom ePRO module (standardized across cancer types) and a customizable disease-specific module. Patient responses feed into a clinician-facing, EHR-embedded clinical decision support component that provides symptom summaries, actionable recommendations, streamlined referrals, and documentation integration to facilitate tailored supportive care delivery. Ongoing monitoring enables longitudinal symptom tracking

To elicit concrete feedback from target users, the research team developed a high-fidelity prototype of the proposed ePRO + CDS system (Fig. 1). Mirroring the visual interface of the institutional electronic health record (EHR), Epic [33], the prototype was developed in a sandbox testing environment in collaboration with EHR analysts to ensure feasibility within available functionality. This EHR-integrated approach incorporated portal-based questionnaire delivery before appointments, point-of-care alerts, and clinical decision support to streamline supportive care referrals based on patient responses. The prototype featured a modular ePRO structure: a core module with proposed use of the Edmonton Symptom Assessment System-Revised (ESAS-r) [34, 35], a validated nine-item measure for common cancer symptoms, complemented by a customizable disease-specific module. For the HNC-specific module, candidate symptoms were drawn from the Patient-Reported Outcomes version of the Common Terminology Criteria for Adverse Events (PRO-CTCAE) item library [36, 37]. Detailed technical specifications of the final system are beyond the scope of this paper.

### Data collection

#### CFIR-guided semi-structured interviews

We conducted CFIR-guided semi-structured interviews with participants between December 2022 and February 2024. Interviews were conducted via teleconference or telephone by trained members of the research team and lasted on average 53 min (range 24–106). Interview guides were developed for each user group, with questions operationalizing CFIR constructs (Supplementary Table S3). All participants provided written informed consent and received a $25 gift card incentive. Interviews were audio-recorded, transcribed verbatim, and de-identified prior to analysis.

During the interviews, participants engaged with the high-fidelity prototype. Clinical staff reviewed clinician-facing displays including symptom summary dashboards and clinical decision support alerts, while patients reviewed the patient portal questionnaire interface. Prototype engagement was interviewer-guided, allowing participants across varying levels of EHR comfort to provide specific input on design preferences, content relevance, and workflow considerations. A key focus was obtaining input on content for the customized module from oncology clinicians, supportive care specialists, and patients. We iteratively incorporated feedback on the relative importance of recommended elements and assessment approaches to ensure the final ePRO content reflected what clinicians needed to know and what patients needed to communicate.

Demographic and professional characteristics were collected from clinical staff. Demographic and patient-reported symptom assessments were collected from patients using the Patient-Reported Outcomes Measurement Information System (PROMIS)-29 Profile version 2.0, which measures physical function, anxiety, depression, fatigue, sleep disturbance, social function, and pain [38].

#### Quantitative pre-implementation measures

Following the interview and prototype demonstration, participants completed a brief quantitative survey assessing anticipated implementation outcomes. These included the Acceptability of Intervention Measure (AIM), Intervention Appropriateness Measure (IAM), and Feasibility of Intervention Measure (FIM), which each include a 4-item scale rated 1 (completely disagree) to 5 (completely agree), with higher scores indicating more favorable perceptions [39]. The survey also included the System Usability Scale (SUS), a 10-item measure scored 0-100, with higher scores indicating better usability [40]. The survey took approximately 5 min and was administered electronically via the Qualtrics XM platform [41].

### Data analysis

#### Qualitative analysis

We used rapid qualitative analysis techniques that are well-suited for implementation research [32, 42], combining directed content analysis guided by CFIR with summarization and matrix analysis [43, 44]. For directed content analysis, a minimum of two trained members of the research team independently coded transcripts using a CFIR-based codebook in Atlas.ti software [45]. The codebook was developed deductively, with CFIR constructs operationalized to the specific context of ePRO implementation for HNC supportive care (Supplementary Table S3). The research team met regularly to discuss analytic decisions and resolve discrepancies through consensus.

Coded data were compiled into construct summaries and organized into matrices to facilitate cross-case comparison and identify patterns, variation across participants and target user groups (clinical staff and patients), and gaps in information. To enhance rigor, the research team documented decisions related to summary and matrix refinements and engaged in reflexive discussions throughout analysis. Summaries and matrices were reviewed in team meetings to discuss emerging patterns and ensure interpretations were grounded in the data.

#### Quantitative analysis

For the quantitative pre-implementation data, we calculated descriptive statistics (means, standard deviations, medians, ranges) for each measure overall and by stakeholder group. We compared ratings between clinical staff and patients using independent samples t-tests with Cohen’s d effect sizes. For PROMIS measures, domain scores were calculated as T-scores standardized to the U.S. general population (mean = 50, SD = 10); physical health and mental health summary scores were generated using factor scoring coefficients [46]. Analyses were conducted using R (version 4.3.1) [47].

## Results

Results are presented in four sections: (1) participant characteristics; (2) CFIR-guided qualitative findings organized by domain and construct; (3) quantitative pre-implementation outcome measures; and (4) integration of findings.

### Participant characteristics

In total, 46 individuals participated: 26 patients (Table 1) and 20 clinical staff (Table 2). Patients (*n* = 26) were predominantly male (73%), White (88%), married (62%), and college-educated (73%), with a mean age of 67 years (SD 9.4). Most (85%) had completed active treatment; treatment modalities included radiation (69.2%), surgery (57.7%), and chemotherapy (34.6%). Most patients (73%) reported frequent patient portal use. Mean PROMIS-29 scores were generally comparable to population norms, though individual scores varied substantially, particularly for fatigue (range 33.7–75.8) and physical function (range 24.6–61.6).

**Table 1:** Patient characteristics

Characteristic	*N* = 26
Demographics	
*Sex*,* n (%)*	
Male	19 (73.1)
Female	5 (19.2)
Not reported	2 (7.7)
*Race*,* n (%)*	
White	23 (88.5)
Asian	2 (7.7)
Black	1 (3.8)
*Marital status*,* n (%)*	
Married/Living as married	16 (61.5)
Divorced/Separated	4 (15.4)
Single (never married)	3 (11.5)
Widowed	1 (3.8)
Not reported	2 (7.7)
*Education*,* n (%)*	
Some high school	1 (3.8)
High school graduate or GED	4 (15.4)
College graduate	14 (53.8)
Graduate degree	5 (19.2)
Not reported	2 (7.7)
*Employment status*,* n (%)*	
Employed	9 (34.6)
Retired	12 (46.2)
Not working due to health	3 (11.5)
Not reported	2 (7.7)
*Annual household income*,* n (%)*	
<$30,000	3 (11.5)
$30,001–$100,000	5 (19.2)
>$100,000	11 (42.3)
Not reported	7 (26.9)
**Clinical Characteristics**	
*Primary insurance*,* n (%)*	
Private insurance	13 (50.0)
Public (Medicare/Medicaid)	11 (42.3)
Not reported	2 (7.7)
Has dental insurance, n (%)	15 (57.7)
*Tumor location*,* n (%)*	
Oral cavity and tongue	13 (50.0)
Oropharynx	3 (11.5)
Larynx	2 (7.7)
Other	8 (30.8)
*Disease stage*,* n (%)*	
Stage I	2 (7.7)
Stage II	4 (15.4)
Stage III	5 (19.2)
Stage IV	5 (19.2)
Unknown	10 (38.5)
Currently receiving treatment, n (%)	2 (7.7)
*Treatments received*,* n (%)*	
Surgery	15 (57.7)
Chemotherapy	9 (34.6)
Radiation therapy	18 (69.2)
Immunotherapy	3 (11.5)
Has caregiver/support person, n (%)	17 (65.4)
Patient portal usage, n (%)	
Always/Almost always	10 (38.5)
Quite a bit	9 (34.6)
Sometimes	5 (19.2)
Occasionally/Rarely/Never	2 (7.7)
**Patient-Reported Outcomes**	
*PROMIS-29 Domain T-scores*,* mean (SD)*	
Physical Function	53.2 (10.6)
Anxiety	50.6 (9.0)
Depression	48.8 (8.2)
Fatigue	49.9 (10.0)
Sleep Disturbance	53.5 (4.2)
Ability to Participate in Social Roles	52.9 (10.6)
Pain Interference	52.4 (10.0)
Pain Intensity	1.8 (1.9)
*PROMIS-29 Summary T-scores*,* mean (SD)*	
Physical Health Summary	53.2 (10.7)
Mental Health Summary	50.3 (8.2)

Abbreviations: PROMIS = Patient-Reported Outcomes Measurement Information System; SD = standard deviation; NRS = numeric rating scale

Note: T-scores are standardized with a mean of 50 and SD of 10 in the U.S. general population. For Physical Function and Ability to Participate in Social Roles, higher scores indicate better functioning. For Anxiety, Depression, Fatigue, Sleep Disturbance, and Pain Interference, higher scores indicate greater symptom severity. Pain Intensity assessed using a single-item 0–10 Numeric Rating Scale, where 0 = no pain and 10 = worst pain

**Table 2:** Clinical staff characteristics

Characteristic	*N* = 20
**Demographic Characteristics**	
Age, years, mean (SD)	44.1 (11.3)
Sex, n (%)	
Female	16 (80.0)
Male	4 (20.0)
Hispanic/Latino ethnicity, n (%)	2 (10.0)
Race, n (%)	
White	11 (55.0)
Asian	8 (40.0)
More than one race	1 (5.0)
**Professional Characteristics**	
Primary clinical role, n (%)	
Physician	9 (45.0)
Nurse/Nurse Practitioner	3 (15.0)
Social Worker/Psychologist	3 (15.0)
Medical Assistant	2 (10.0)
Administrator	1 (5.0)
Rehabilitative Specialists	2 (10.0)
Highest education level, n (%)	
Doctoral degree	11 (55.0)
Master’s degree	6 (30.0)
Bachelor’s degree	2 (10.0)
Vocational/technical training	1 (5.0)
Years at current institution, mean (SD)	9.0 (8.3)
Years working with HNC patients, mean (SD)	10.1 (11.5)
Proportion of clinical practice with HNC patients, n (%)	
0–25%	6 (30.0)
26–50%	3 (15.0)
51–75%	5 (25.0)
76–100%	6 (30.0)

Clinical staff (*n* = 20) were predominantly female (80%) with a mean age of 44.1 years (SD 11.3) and 10.1 years on average of clinical HNC experience (SD 11.5) (Table 2). Clinical staff participants represented oncology subspecialties including medical oncology (*n* = 5), radiation oncology (*n* = 3), head and neck surgery (*n* = 3), nursing (*n* = 3), and supportive care specialists (*n* = 6) including speech-language pathology, physical therapy, nutrition, social work, psycho-oncology, and palliative care.

### CFIR-guided qualitative findings

Key qualitative findings were identified within five CFIR constructs across three domains: Inner Setting (Tension for Change, Compatibility), Innovation (Design, Relative Advantage), and Individuals (Needs, Opportunity, and Motivation) (Table 3).

**Table 3:** CFIR-guided qualitative findings: key themes by domain, construct, and user group

CFIR Domain	Construct	Operational Definition	User Group	Key Findings	Exemplar Quotes
Inner Setting	Tension for Change	Degree to which target users perceive current situation as needing change	Clinical Staff	• Current symptom assessment ad hoc and variable • Incomplete and unsystematic picture of patient symptoms • Late referrals limiting intervention potential	*“Most of our referrals for symptom management are really triggered quite informally.” (Staff 5)* *“Whoever is in a position to potentially place that referral*,* it tends to often be just comfort level*,* not so much based on stage of disease or symptom burden.” (Staff 14)* *“I get [the patients] a little late. And I’m playing catch up. And I’m like*,* okay*,* we’re gonna do the best we can here.” (Staff 17)* *“There’s a whole range of people not being captured and not being referred to us. And it’s really critical in speech and swallowing that they’re seen earlier.” (Staff 13)*
			Patients	• Haphazard current approach • Need to prepare for appointments • Missed opportunities for early support; preventable harm from delays	*“I definitely think post-treatment follow up - to be a little more proactive managing post-treatment is where the greatest room for improvement is.” (Patient 14)* *“You’ve got to go in prepared because the doctors don’t have enough time.” (Patient 25)* *“You go in with a lot going on*,* and it’s easy to forget things you’re experiencing.” (Patient 4)*
	Compatibility	Degree to which innovation fits with existing workflows, systems, and values	Clinical Staff	• Facilitators: EHR-native design; aligns with existing momentum • Concerns: Alert fatigue; downstream capacity, barriers	*“It’d be really great to have all this information before seeing a patient in clinic. As part of a clinic visit. Seems like it’s easy to get into a note. It’s easy to track trends.” (Staff 3)* *“Adopting this kind of thing very easily overwhelms your clinic. And if patients raise the issue*,* and you don’t address it*,* it looks like bad medicine.” (Staff 7)* *“Potential for survey fatigue*,* potential for noncompliance*,* potential for them not having MyChart*,* and then languages.” (Staff 3)*
			Patients	• Familiarity with EHR patient portal • Portal as trusted communication tool	*“The portal is great and easy to navigate whether it’s on my computer or my phone.” (Patient 5)* *“If I have a question*,* it’s going to be through [the portal] because I love that tool*,* very honestly. I think it’s very efficient. And I know that all the staff is incredibly busy so it’s hard to get access. I think that’s the most efficient way to go.” (Patient 23)* For patients with treatment-related communication impairments, verbal reporting posed additional obstacles: *“I think it’s a need actually*,* especially for those moments where you really can’t verbalize.” (Patient 11)*
Innovation	Design	How the innovation is designed, bundled, packaged	Clinical Staff	• Reduced searching • Soft prompts over hard stops	*“It would help to have it more focused on what we’re looking for*,* so I don’t have to search” (Staff 11)* *“Once it prompts [the clinicians]*,* that serves as a good reminder. The buttons just make it so much easier” (Staff 4)*
			Patients	• Simplicity, clarity, brevity prioritized • Intuitive navigation; Mobile-friendly • Balance comprehensiveness and relevance with low burden	*“You don’t want to make it difficult*,* then people won’t do it.” (Patient 12)* *“It’s very clear and straightforward to answer.” (Patient 17)* *“It’s hard for you to think of all the things*,* so these are essentially prompts -- that’s right*,* I do have the change in my taste.” (Patient 14)* *“The questions are easy… ‘In the last seven days’… that’s important because things could have changed from the last one.” (Patient 7)*
	Relative Advantage	Target users’ perceived benefit of innovation relative to alternate approaches	Clinical Staff	• ePRO + CDS perceived as enabling proactive, whole‑person care • Scalability beyond HNC • Linked CDS as key advantage	*“This information will shift providers’ perspective to be thinking of their patients as whole people.” (Staff 2)* *“I like this because it’s patient-reported and patient-centered… it’s a place to start a discussion of what’s most important.” (Staff 1)* *“To pull it into my note would be great. I can see what’s positive and negative and deal with it.” (Staff 17)*
			Patients	• Proactive vs. reactive care • Validation of experiences • Care transformation	*“Forewarned is forearmed—they know what’s going on before I walk in.” (Patient 2)* *“It brings the communication between the doctor and the patient. […] They’re going to see my answers and know what my thoughts and feelings and pains and aches are. So the communication is already established and things won’t be overlooked.” (Patient 8)* *“[The tool] validates when these things happen. That this is part of the disease treatment*,* and it would put some normalcy to it all. […] A lot of times you don’t know what questions to even ask. When I got neuropathy in my feet I didn’t know what that was… To normalize if you’re having weird symptoms I think helps too.” (Patient 23)*
Individuals	Needs, Opportunity, and Motivation	Requirements that influence whether individuals engage with the innovation	Innovation Deliverers (Clinical Staff)	• Structured recommendations for clinical response • Symptom-specific routing • Tiered response model	*“You need to know what to ask*,* when to ask it. Having this formalized in the electronic records would be the much better approach.” (Staff 7)* *“If a person was answering a specific way*,* it popped up: ‘Consider dietitian referrals’… this is wonderful.” (Staff 13)*
			Innovation Recipients (Patients)	• Data must be used by providers • Skepticism about clinician engagement • Need for clear purpose	*“If everyone uses this it’s going to help*,* but the doctors have to show the patient that they’re using this. The more they show that*,* the more patients will use it.” (Patient 5)* *“If you can get the doctors to read it – which I would bet you that 50% wouldn’t even look at it – it’s like you fill out all this stuff and you’re doing it for the lawyers*,* not doctors. [The portal’s] only as good as what they’re going to use it for.” (Patient 25)* *“You can give people the best tools in the world*,* but if they’re not incentivized to use them—that’s the elixir*,* that’s the silver bullet.” (Patient 16)*

#### Inner setting: tension for change

Tension for change reflects target users’ perceived need for change from current practices. Both clinical staff and patients expressed strong tension for change regarding symptom assessment and supportive care delivery. Clinical staff characterized current approaches to symptom assessment as unsystematic and variable, with one clinician describing an “unofficial triage system” (Staff 7) where only the most severe symptoms receive attention and only when patients raise them. Multiple clinicians described ad hoc practices: “We don’t have formal scales, we don’t have patients fill anything out, and we don’t track it in a systematic way” (Staff 5). Another concluded: “We really don’t have a very fine-grained picture of what a lot of patients go through” (Staff 2). Variation across providers was also acknowledged: “It often is not dependent on anything related to the patient, but it’s very physician dependent. […] There are physicians that essentially will never refer, even in cases that […] would be very, very likely to benefit” (Staff 14). Supportive care specialists expressed frustration with late referrals, often received after functional losses had become difficult to reverse. One specialist described repeatedly wondering, “Why didn’t I see them earlier?” (Staff 17).

Patients described the challenge of communicating multiple symptoms during brief appointments, noting they often feel overwhelmed or forget concerns in the moment: “a lot of people go in there and they just either get overwhelmed or forget a lot of stuff that they’re experiencing, and the doctor can’t help them if they don’t know about it” (Patient 1). Others noted that patients may not recognize their symptoms as related to their cancer, and so may not report them: “You might not even realize these symptoms are symptoms because you think they’re your new normal, but they’re not normal” (Patient 16). Without systematic approaches, patients described missed opportunities for support and preventable harm. One recounted: “I honestly was discouraged a lot because I was feeling like no one could help me. […] I had to just live with it” (Patient 1). Another patient shared: “Nobody really saw me until after I was finished with chemo and radiation, when the damage was done” (Patient 13).

#### Inner setting: compatibility

Compatibility reflects the fit between the innovation and existing workflows, systems, and values. For clinical staff, EHR integration was a key facilitator: “We all live in the EHR and that’s where the information needs to be” (Staff 3). Others noted alignment with existing momentum: “Our departments are already moving towards something like this - we’re big fans” (Staff 7). Despite overall enthusiasm, some oncology staff expressed concerns regarding downstream capacity and the need for actionable interventions, with one explaining, “There’s no way I can address that in a meaningful way” (Staff 5). Another provider articulated the tension: “If we screen for it and don’t have anything to offer for it, then that’s maybe not good” (Staff 12). Alert fatigue was also widely acknowledged: “Best practice alert [BPA] fatigue is very real. I just click through these” (Staff 9). Notably, supportive care specialists did not share capacity concerns, viewing under-referral as the more pressing problem.

Patients perceived the platform as highly compatible with existing routines. Most had already invested considerable effort in learning the patient portal, with many noting it was the most direct way to access their care team. Integrating symptom assessments into a platform they already use was seen as a natural fit. “[The portal] is what I use for everything - appointments, messages, test results” (Patient 5). Pre-visit timing was generally viewed as “perfectly logical” (Patient 20). However, one patient raised the issue of optimal timing in the treatment trajectory, explaining, “At the very beginning, I didn’t have a lot of these… so having this before might be really overwhelming… but later on, you could relate to everything here” (Patient 21).

#### Innovation: design

Design reflects how the innovation is assembled, bundled, and presented. Clinical staff articulated specific design requirements, emphasizing integration and minimal added burden. Streamlined ordering for existing but difficult-to-locate e-referrals and integration with note templates via CDS were viewed as significant advantages: “The buttons just make it so much easier…’Do you want this referral? Click all that apply.’ How easy is that?” (Staff 4). Providers emphasized the challenge of locating information within the electronic chart and the need for prominent visibility: “If I have to search for it, I probably won’t do anything about it” (Staff 8). Visual hierarchy to signal severity was strongly recommended: “Make it red text or something, so providers’ eyes are drawn to it” (Staff 3). Providers generally preferred passive alerts over hard stops to minimize workflow disruption.

Patients emphasized simplicity and ease of use, with one cautioning: “If it’s complicated and long the patient’s not going to put any effort in it and the doctor’s not going to read it” (Patient 25). After reviewing the prototype, patients praised the interface as comprehensive yet accessible: “[It] addressed literally every issue I had and was very easy to navigate” (Patient 11). Another reiterated: “I don’t know how much more simple it could be” (Patient 5). Several also valued the privacy and reflection the format offered: “Sometimes these questions get a little personal, and being able to check it off in your own privacy is a huge benefit” (Patient 26). As another explained, it “gives patients time to really think about answering those questions rather than just doing it in front of the doctor” (Patient 20). Patients recommended assessments take less than five minutes, avoid jargon, and be phone accessible.

#### Innovation: relative advantage

Relative advantage reflects target users’ perception of benefit compared to alternative approaches. Clinical staff perceived strong relative advantage, describing the system as having “potential to be transformative in terms of care” (Staff 2). Providers appreciated that the tool’s comprehensive scope supported “a whole person approach, which in the context of cancer care, is fundamental to ensuring that we’re providing quality care” (Staff 19). Unlike standalone platforms, the system’s seamless EHR integration addressed the common frustration of logging into yet another system. Beyond accessibility, clinical staff valued how the system enabled systematic, proactive symptom identification across the multidisciplinary team. The tailored CDS recommendations were particularly valued, with one clinician stating: “If [patients] can screen and say, Hey, I’m having these issues and [oncologists] can say, hey, there’s help, this is who’s appropriate to help you with this. That’s a dream come true” (Staff 18). The combination of clinical utility and workflow integration also offered efficiency gains, with staff perceiving the tool’s functionality as a documentation “time-saver” (Staff 12). Finally, the potential for scalability with the modular design was recognized as “broadly applicable to all the different disease sites” (Staff 5).

Patients also perceived considerable advantages over current approaches. They recognized that a symptom checker could focus brief appointments: “Sometimes when you go in you forget what you want to ask. The doctors are on kind of a strict timeline, too. They can’t cover every question” (Patient 14). Another explained that the tool could “eliminate a lot of fact-finding time” and “get right to the point of what’s bothering that patient symptom-wise. It helps focus the visit on what actually matters” (Patient 4).

Patients felt the proactive approach might reduce anxiety and improve their understanding of their symptoms. One patient explained, “If you’re forewarning the patient, that takes a lot of the fear away, creates a comfort for a patient” (Patient 26). Several described how the questionnaires could normalize symptom experiences: “Even just the process of answering these questions, you feel sort of validated” (Patient 11). As with clinical staff, patients perceived the ability to link symptoms to resources as transformative: “The ability to pair up medical help with those [symptom] categories…I think that’s just a revolutionary concept” (Patient 26).

#### Individuals: needs, opportunity, and motivation

Needs, opportunity, and motivation reflect requirements influencing engagement among both clinical staff (innovation deliverers) and patients (innovation recipients). Clinical staff emphasized that ePRO screening alone, without CDS, was insufficient, but a screening tool linked with clear guidance for clinical action would be “a god send” (Staff 18). Another reiterated: “A screening tool itself is not going to be nearly as impactful as a screening tool with very clear guidance as to what one should do with that information” (Staff 14). Providers articulated preferences for logic-driven CDS including symptom-specific referral routing (“voice problem, dysphonia, speech problem, and swallowing are all different”) and threshold-based prompting with tiered responses (Staff 13). One provider explained how these elements could help “catch people sooner, so we can do something” (Staff 17), before symptoms progress and rehabilitation potential is limited. Clinical staff also recognized patient needs beyond the proposed system’s scope, particularly social determinants of health. One provider shared a challenging case of an unhoused patient with a g-tube after completing chemoradiation: “We seem to be honing in on the physical and the mind. There’s a whole other section of this that isn’t really being addressed” (Staff 10).

Patient engagement was contingent on clinician use of the data. Several patients expressed enthusiasm about the innovation concept but skepticism about their clinical teams: “I would bet you that 50% [of oncologists] wouldn’t even look at it” (Patient 25). Others emphasized that communicating a clear benefit would be a strong motivator: “I want to know why are you making me do this” (Patient 20). Patients indicated that demonstrated value would sustain engagement: “If I knew that they were definitely going to look at this and act upon it, absolutely. All it takes is that one time for me to realize they are doing it for me to continue” (Patient 5). For some, oncologist involvement was essential: “I’d want to know that the doctor, specifically the doctor, sees the answers” (Patient 12). These findings highlight that successful implementation requires closed-loop care, equipping clinicians to act on patient-reported data and demonstrating to patients that their input informs care.

### Quantitative pre-implementation measures

After engaging with the prototype, participants completed quantitative measures assessing acceptability, appropriateness, feasibility, and usability (Table 4). Both clinical staff and patients rated the proposed ePRO system highly across all metrics. Mean scores on the AIM, IAM, and FIM exceeded 4.0 for both groups, indicating strong pre-implementation readiness. Mean SUS scores fell within the “excellent” range (> 80) for both groups. Independent samples t-tests revealed no statistically significant differences between clinical staff and patients on any measure (all *p* > .05), indicating alignment across user groups.

**Table 4:** Pre-implementation acceptability, appropriateness, feasibility, and usability by user group

Measure	Clinical Staff (*n* = 20)	Patients (*n* = 26)	t	*p*	Cohen’s d
	Mean (SD)	Mean (SD)			
Acceptability (AIM)	4.47 (0.48)	4.21 (0.62)	1.60	0.116	0.46
Appropriateness (IAM)	4.46 (0.59)	4.24 (0.63)	1.22	0.230	0.36
Feasibility (FIM)	4.41 (0.51)	4.35 (0.56)	0.38	0.707	0.11
System Usability Scale (SUS)	82.2 (14.5)	85.6 (13.1)	-0.82	0.416	-0.25

Note. AIM, IAM, and FIM scored on 5-point Likert scale (1 = completely disagree to 5 = completely agree); SUS scored 0-100, with higher scores indicating better usability

### Integration of findings

Findings demonstrated strong support for the proposed ePRO + CDS system, with qualitative themes aligning with quantitative metrics. Across both data sources, actionable clinical decision support emerged as a critical requirement. Patients emphasized that sustained engagement required evidence of provider response: “If I knew [my providers] were definitely going to look at this and act upon it, absolutely” (Patient 5). Clinical staff concurred, describing ePRO data collection without CDS as “pointless” (Staff 17). This convergence underscores that closing the feedback loop between patient report and clinical action is critical to implementation success. Table 5 presents key design priorities and implementation strategies.

**Table 5:** Key design priorities and implementation strategies derived from target user input

	Findings
**DESIGN PRIORITIES**	
EHR Integration	• Full EHR integration with no manual workflows or stand-alone systems • Leverage native EHR functionality, including patient portal
ePROs With an Action Plan	• Actionable ePROs with clear purpose; no data collection without clinical response • Tailored recommendations linking patient responses to supportive care referrals
Simple Design for Real-World Use	• Modular framework for scalability • Simple, visual clinician interface • Brief patient questionnaires
**IMPLEMENTATION STRATEGIES**	
Education & Training	• Train all staff and develop training materials • Facilitation and ongoing support during rollout
Support Clinical Workflow	• No pressure to pre-review before appointments • Prioritize visual impact; minimize extra clicks
Phased Approach	• Start focused, expand systematically • Monitor engagement through regular reports • Iterative refinement based on user feedback
Leadership & Infrastructure	• Secure IT and leadership commitments • Leverage existing infrastructure to enable integration

## Discussion

This CFIR-guided pre-implementation formative evaluation revealed strong target user support for implementing an ePRO-based symptom screening and clinical decision support system in head and neck cancer care. Both clinicians and patients identified substantial tension for change, describing current symptom assessment as “haphazard,” leading to late or missed supportive care referrals and the potential for preventable suffering. Target users endorsed the proposed intervention’s compatibility with existing workflows while providing specific guidance on design attributes to optimize integration. Quantitative pre-implementation metrics indicated strong readiness, with excellent ratings across acceptability, appropriateness, feasibility, and usability. These findings extend prior research on gaps in systematic symptom assessment,^6,16^ while providing compelling evidence of the clinical consequences from the perspectives of patients describing functional loss and supportive care specialists highlighting missed opportunities for earlier intervention.

A central theme was the need for actionable clinical guidance embedded in the EHR. Clinical staff described data collection without actionable guidance as “pointless,” while patients made clear that their sustained engagement depended on meaningful provider response. A growing body of evidence from research trials demonstrates that while ePRO monitoring can improve certain outcomes, structured clinical response may optimize these benefits. In an early symptom monitoring trial without CDS, oncology providers received over 1,000 alerts – nearly half at severe levels – yet initiated only 20 patient contacts, and symptom outcomes did not improve [48]. When the same system was paired with nurse practitioners using guideline-based decision support, symptom severity decreased by 43% and severe symptom days by 67% [49]. The recent PRO-TECT trial demonstrated quality of life benefits and reduced emergency visits using a monitoring plus alerts model, but operated outside the EHR with clinical response left to provider discretion – factors the authors acknowledged may have limited impact on other outcomes such as survival [50]. The emphasis on full EHR integration in our study is consistent with recent ePRO guidance, warning that standalone systems disconnected from the EHR face significant adoption barriers [15, 51].

Real-world implementation evidence reinforces these concerns. The Symptom Management Implementation of Patient-Reported Outcomes in Oncology (SIMPRO) consortium developed and deployed eSyM, an EHR-integrated ePRO system available through Epic, in patients with gastrointestinal, gynecologic, and thoracic malignancies across six U.S. cancer centers [52–54]. Over 22,000 patients used eSyM between 2019 and 2023, with preliminary data showing reduced emergency visits and hospitalizations among symptom responders. Despite full EHR integration, the system relied on designated staff pools to respond to alerts without structured clinical guidance. Recent reviews caution that alert-only models are insufficient when clinicians lack clear pathways to act—whether due to inadequate training, limited referral options, or unclear intervention criteria [14, 50, 53–55].

In another example, the PROMPT-Care study implemented ePROs in routine lung cancer care using alert-driven workflows managed by care coordinators, but without CDS embedded at the point of care [55]. Although nearly all alerts were actioned and patients received significantly more supportive care referrals than historical controls, this workflow operated largely in parallel to oncology appointments. While oncologists did receive summaries or ePROs and related follow-up, they were effectively outside the workflow and did not routinely engage with the ePRO data during patient encounters. Without a clear connection between their reported symptoms and clinical response, patient engagement declined substantially, suggesting that patients need to perceive ePRO completion as direct communication with their clinical team.

Many existing models rely on dedicated personnel such as nurses, care coordinators, or patient navigators to respond to alerts [50, 52, 55]. While potentially effective, this approach is resource-intensive, vulnerable to staffing disruptions, and difficult to sustain or scale. Our model addresses this by embedding CDS directly into the EHR, enabling multiple clinicians across the multidisciplinary team to act on patient-reported data through structured recommendations aligned with available supportive care resources. Planned implementation strategies include training clinicians to not only use the workflow, but explicitly reference ePRO findings during encounters, reinforcing the connection between patient-reported data and clinical response.

A distinctive feature of our approach is the modular architecture pairing assessment of core symptoms with customizable disease-specific screening, each linked to tailored clinical decision support. Many approaches apply generic measures across cancer populations, capturing common symptoms (e.g., fatigue, pain), but missing disease-specific concerns [50, 53, 54]. In the use case of HNC, these may include issues related to swallowing, speech, and dental function. Patients valued that the prototype “addressed literally every issue that I had,” suggesting that disease-specific content enhances relevance and may also help sustain engagement. The design allows for adaptation to other cancers without rebuilding the system, positioning this approach for scalable, disease-tailored implementation.

These findings should be interpreted in light of several limitations. This evaluation occurred at a single academic center with robust EHR infrastructure and high baseline portal adoption; findings may not transfer to community settings with fewer resources or lower portal use. The patient sample skewed toward White, college-educated individuals, limiting transferability to more diverse populations, though some evidence suggests populations with limited computer experience may derive greater benefit from ePRO programs [15, 56]. Although prototype engagement did not require independent portal use, patients who chose to participate may have been more engaged with their care or more comfortable with electronic monitoring. Our HNC focus may also limit transferability, though the design is structured for customization and scalability across disease sites. Future research should evaluate implementation and clinical outcomes in more diverse and resource-varied settings and examine the adaptation process to other cancer populations.

## Conclusions

This pre-implementation formative evaluation demonstrates the value of systematic target user engagement and implementation science frameworks in the ePRO discovery phase. Key findings include the importance of EHR integration, actionable clinical decision support, and the potential scalability of the modular design. Target users emphasized the inadequacy of ePRO monitoring without structured clinical response, underscoring closed-loop care as a key determinant for successful implementation. By embedding structured CDS and referral pathways into EHR architecture, the proposed ePRO + CDS system is positioned as a scalable model for real-world implementation to promote delivery of supportive care.

## Supplementary Information

Below is the link to the electronic supplementary material.

Supplementary Material 1

## Data Availability

The deidentified datasets used and/or analyzed during the current study are available from the corresponding author on reasonable request.

## References

[1] Rodin G, Feldman A, Trapani D et al (2025) The human crisis in cancer: a Lancet Oncology Commission. Lancet Oncol 26(12):e628–e670. 10.1016/S1470-2045(25)00530-3 41192457

[2] Chan RJ, Knowles R, Ashbury FD et al (2024) Supportive care 2030 movement: towards unifying ambitions for global excellence in supportive cancer care—an international Delphi study. EClinicalMedicine 76. 10.1016/j.eclinm.2024.102825 PMC11415959 39309723

[3] Hui D (2014) Definition of supportive care. Curr Opin Oncol 26(4):372–379. 10.1097/CCO.0000000000000086 PMC12034444 24825016

[4] Wagle NS, Nogueira L, Devasia TP et al (2025) Cancer treatment and survivorship statistics, 2025. CA Cancer J Clin 75(4):308–340 .70011;CTYPE:STRING:JOURNAL 10.3322/caac.70011 PMC12223361 40445120

[5] Balitsky AK, Rayner D, Britto J et al (2024) Patient-Reported Outcome Measures in Cancer Care: An Updated Systematic Review and Meta-Analysis. JAMA Netw Open 7(8):e2424793–e2424793. 10.1001/JAMANETWORKOPEN.2024.24793 PMC11322847 39136947

[6] Lai-Kwon J, Rutherford C, Jefford M, Gore C, Best S (2024) Using Implementation Science Frameworks to Guide the Use of Electronic Patient-Reported Outcome Symptom Monitoring in Routine Cancer Care. JCO Oncol Pract 20(3):335–349. 10.1200/OP.23.00462 38206290

[7] Atkinson TM, Ryan SJ, Bennett AV et al (2016) The association between clinician-based common terminology criteria for adverse events (CTCAE) and patient-reported outcomes (PRO): a systematic review. Supportive Care Cancer Springer Verlag 24(8):3669–3676. 10.1007/s00520-016-3297-9 PMC4919215 27260018

[8] Laugsand EA, Sprangers MAG, Bjordal K, Skorpen F, Kaasa S, Klepstad P (2010) Health care providers underestimate symptom intensities of cancer patients: A multicenter European study. Health Qual Life Outcomes 8(1):104. 10.1186/1477-7525-8-104 PMC2949821 20858248

[9] Chow R, Zimmermann C, Bruera E, Temel J, Im J, Lock M (2020) Inter-rater reliability in performance status assessment between clinicians and patients: a systematic review and meta-analysis. BMJ Support Palliat Care 10(2):129–135. 10.1136/BMJSPCARE-2019-002080 31806655

[10] Fromme EK, Eilers KM, Mori M, Hsieh YC, Beer TM (2004) How accurate is clinician reporting of chemotherapy adverse effects? A comparison with patient-reported symptoms from the Quality-of-Life Questionnaire C30. J Clin Oncol 22(17):3485–3490. 10.1200/JCO.2004.03.025 15337796

[11] Minello C, George B, Allano G, Maindet C, Burnod A, Lemaire A (2019) Assessing cancer pain—the first step toward improving patients’ quality of life. Support Care Cancer 27(8):3095–3104. 10.1007/s00520-019-04825-x 31076899

[12] Basch E, Barbera L, Kerrigan CL, Velikova G (2018) Implementation of Patient-Reported Outcomes in Routine Medical Care. Am Soc Clin Oncol Educ Book 38(38):122–134. 10.1200/EDBK_200383 30231381

[13] Kaufmann TL, Rocque GB (2024) Using Electronic Patient-Reported Outcome Monitoring to Navigate Patients to Supportive Care Services. JCO Oncol Pract 20(10):1297–1299. 10.1200/OP.24.00270 PMC13040481 38917403

[14] Stout NL, Alfano CM, Liu R, Dixit N, Jefford M (2024) Implementing a Clinical Pathway for Needs Assessment and Supportive Care Interventions. JCO Oncol Pract 20(9):1173–1181. 10.1200/OP.23.00482 PMC11572702 38709984

[15] Basch E, Rocque G, Mody G, Mullangi S, Patt D (2023) Tenets for implementing electronic patient-reported outcomes for remote symptom monitoring during cancer treatment. JCO Clin Cancer Inf 7(7). 10.1200/CCI.22.00187 PMC11770894 36857630

[16] Di Maio M, Basch E, Denis F et al (2022) The role of patient-reported outcome measures in the continuum of cancer clinical care: ESMO Clinical Practice Guideline ☆. Ann Oncol 33(9):878–892. 10.1016/j.annonc.2022.04.007 35462007

[17] Mooney K, Gullatte M, Iacob E et al (2024) Essential Components of an Electronic Patient-Reported Symptom Monitoring and Management System: A Randomized Clinical Trial. JAMA Netw Open 7(9):e2433153. 10.1001/JAMANETWORKOPEN.2024.33153 PMC11400212 39269704

[18] Hui D, Subbiah IM, Hong D et al (2025) Integration of remote monitoring into palliative care for patients with advanced cancer undergoing phase I therapies: a randomized clinical trial. JCO Oncol Pract Published online September 23. 10.1200/OP-25-00442 40986798

[19] Basch E, Hudson K, Rocque G (2023) Implementation of electronic patient-reported outcomes for symptom monitoring during cancer treatment: the importance of getting it right. J Comp Eff Res 12(12):e230157. 10.57264/CER-2023-0157 PMC10734319 37961992

[20] Damschroder LJ, Aron DC, Keith RE, Kirsh SR, Alexander JA, Lowery JC (2009) Fostering implementation of health services research findings into practice: a consolidated framework for advancing implementation science. Implement Sci 4(1):50. 10.1186/1748-5908-4-50 PMC2736161 19664226

[21] Damschroder LJ, Reardon CM, Widerquist MAO, Lowery J (2022) The updated consolidated framework for implementation research based on user feedback. Implement Sci 17(1):1–16. 10.1186/S13012-022-01245-0 PMC9617234 36309746

[22] Damschroder LJ (2020) Clarity out of chaos: Use of theory in implementation research. Psychiatry Res 283:112461. 10.1016/J.PSYCHRES.2019.06.036 31257020

[23] Stetler CB, Legro MW, Wallace CM et al (2006) The role of formative evaluation in implementation research and the QUERI experience. J Gen Intern Med 21(2). 10.1111/J.1525-1497.2006.00355.X PMC2557128 16637954

[24] Ringash J, Bernstein LJ, Devins G et al (2018) Head and Neck Cancer Survivorship: Learning the Needs, Meeting the Needs. Semin Radiat Oncol 28(1):64–74. 10.1016/j.semradonc.2017.08.008 29173757

[25] Murphy BA, Wulff-Burchfield E, Ghiam M, Bond SM, Deng J (2019) Chronic systemic symptoms in head and neck cancer patients. J Natl Cancer Inst Monogr 2019(53). 10.1093/JNCIMONOGRAPHS/LGZ004 31425597

[26] Nguyen NTA, Ringash J (2018) Head and neck cancer survivorship care: a review of the current guidelines and remaining unmet needs. Curr Treat Options Oncol 19(8). 10.1007/s11864-018-0554-9 29987676

[27] Ringash J (2015) Survivorship and quality of life in head and neck cancer. Journal Clin Oncology American Soc Clin Oncology 33(29):3322–3327. 10.1200/JCO.2015.61.4115 26351336

[28] Chera BS, Eisbruch A, Murphy BA et al (2014) Recommended patient-reported core set of symptoms to measure in head and neck cancer treatment trials. J Natl Cancer Inst 106(7). 10.1093/jnci/dju127 PMC4192043 25006189

[29] Tong A, Sainsbury P, Craig J (2007) Consolidated criteria for reporting qualitative research (COREQ): a 32-item checklist for interviews and focus groups. Int J Qual Health Care 19(6):349–357. 10.1093/INTQHC/MZM042 17872937

[30] Pinnock H, Barwick M, Carpenter CR et al (2017) Standards for reporting implementation studies (StaRI) Statement. BMJ 356. 10.1136/BMJ.I6795 PMC5421438 28264797

[31] Palinkas LA, Horwitz SM, Green CA, Wisdom JP, Duan N, Hoagwood K (2015) Purposeful sampling for qualitative data collection and analysis in mixed method implementation research. Adm Policy Ment Health 42(5):533. 10.1007/S10488-013-0528-Y PMC4012002 24193818

[32] Hamilton AB, Finley EP (2019) Qualitative methods in implementation research: An introduction. Psychiatry Res 280. 10.1016/J.PSYCHRES.2019.112516 PMC7023962 31437661

[33] Epic (2026) With the patient at the heart. Accessed June 8. https://www.epic.com/

[34] Watanabe SM, Nekolaichuk C, Beaumont C, Johnson L, Myers J, Strasser F (2011) A multicenter study comparing two numerical versions of the Edmonton symptom assessment system in palliative care patients. J Pain Symptom Manage 41(2):456–468. 10.1016/j.jpainsymman.2010.04.020 20832987

[35] Hui D, Bruera E (2017) The Edmonton Symptom Assessment System 25 Years Later: Past, Present, and Future Developments. J Pain Symptom Manage 53(3):630–643. 10.1016/j.jpainsymman.2016.10.370 PMC5337174 28042071

[36] The PRO-CTCAE Measurement System. Accessed August 21 (2022) https://healthcaredelivery.cancer.gov/pro-ctcae/measurement.html

[37] Dueck AC, Mendoza TR, Mitchell SA et al (2015) Validity and Reliability of the US National Cancer Institute’s Patient-Reported Outcomes Version of the Common Terminology Criteria for Adverse Events (PRO-CTCAE). JAMA Oncol 1(8):1051–1059. 10.1001/jamaoncol.2015.2639 PMC4857599 26270597

[38] Cella D, Riley W, Stone A et al (2010) The patient-reported outcomes measurement information system (PROMIS) developed and tested its first wave of adult self-reported health outcome item banks: 2005–2008. J Clin Epidemiol 63(11):1179–1194. 10.1016/J.JCLINEPI.2010.04.011 PMC2965562 20685078

[39] Weiner BJ, Lewis CC, Stanick C et al (2017) Psychometric assessment of three newly developed implementation outcome measures. Implement Sci 12(1):1–12. 10.1186/S13012-017-0635-3/TABLES/3 PMC5576104 28851459

[40] Brooke J (1996) SUS-A Quick and Dirty Usability Scale. Accessed October 3, 2019. https://www.TBIStaffTraining.info

[41] Qualtrics XM. https://www.qualtrics.com

[42] Nevedal AL, Reardon CM, Opra Widerquist MA et al (2021) Rapid versus traditional qualitative analysis using the consolidated framework for implementation research (CFIR). Implement Sci 16(1). 10.1186/S13012-021-01111-5 PMC8252308 34215286

[43] Hsieh HF, Shannon SE (2005) Three approaches to qualitative content analysis. Qual Health Res 15(9):1277–1288. 10.1177/1049732305276687 16204405

[44] Gale NK, Heath G, Cameron E, Rashid S, Redwood S (2013) Using the framework method for the analysis of qualitative data in multi-disciplinary health research. BMC Med Res Methodol 13(1). 10.1186/1471-2288-13-117 PMC3848812 24047204

[45] ATLAS.ti Scientific Software Development GmbH. [Qualitative data analysis software]. Scientific Software Development. Preprint posted online 2023:v9.15.3-2025-04-06. Accessed April 7 (2025) https://atlasti.com

[46] Hays RD, Spritzer KL, Schalet BD, Cella D (2018) PROMIS^®^-29 v2.0 Profile Physical and Mental Health Summary Scores. Qual Life Res 27(7):1885. 10.1007/S11136-018-1842-3 PMC5999556 29569016

[47] R Core Team R: A language and environment for statistical computing. R Foundation for Statistical Computing. Preprint posted online 2021:1.3.1073

[48] Mooney KH, Beck SL, Friedman RH, Farzanfar R, Wong B (2014) Automated monitoring of symptoms during ambulatory chemotherapy and oncology providers’ use of the information: a randomized controlled clinical trial. Support Care Cancer 22(9):2343. 10.1007/S00520-014-2216-1 PMC4290846 24687538

[49] Mooney KH, Beck SL, Wong B et al (2017) Automated home monitoring and management of patient-reported symptoms during chemotherapy: results of the symptom care at home RCT. Cancer Med 6(3):537–546. 10.1002/CAM41002;REQUESTEDJOURNAL:JOURNAL:20457634;WGROUP:STRING:PUBLICATION PMC5345623 28135050

[50] Basch E, Schrag D, Jansen J et al (2025) Symptom monitoring with electronic patient-reported outcomes during cancer treatment: final results of the PRO-TECT cluster-randomized trial. Nat Med 31(4):1225–1232. 10.1038/S41591-025-03507-Y PMC12184200 39920394

[51] Nguyen H, Butow P, Dhillon H, Sundaresan P (2021) A review of the barriers to using Patient-Reported Outcomes (PROs) and Patient-Reported Outcome Measures (PROMs) in routine cancer care. J Med Radiat Sci 68(2):186–195. 10.1002/JMRS.421 PMC8168064 32815314

[52] Hassett MJ, Wong S, Osarogiagbon RU et al (2022) Implementation of patient-reported outcomes for symptom management in oncology practice through the SIMPRO research consortium: a protocol for a pragmatic type II hybrid effectiveness-implementation multi-center cluster-randomized stepped wedge trial. Trials 23(1). 10.1186/S13063-022-06435-1 PMC9202326 35710449

[53] Paudel RMNHMMAK (2025) Unlocking the potential of patient-reported outcomes: practical uses in routine oncology care. Clin Adv Hematol Oncol 23(8):512–519 41236431

[54] Hassett MJ, Dias S, Cronin C et al (2024) Strategies for implementing an electronic patient-reported outcomes-based symptom management program across six cancer centers. Res Sq Published online January 24. 10.21203/RS.3.RS-3879836/V1 PMC11558981 39533260

[55] Girgis A, Bamgboje-Ayodele A, Rincones O et al (2022) Stepping into the real world: a mixed-methods evaluation of the implementation of electronic patient reported outcomes in routine lung cancer care. J Patient Rep Outcomes 6(1). 10.1186/S41687-022-00475-6 PMC9207870 35723827

[56] Basch E, Deal AM, Kris MG et al (2016) Symptom Monitoring With Patient-Reported Outcomes During Routine Cancer Treatment: A Randomized Controlled Trial. J Clin Oncol 34(6):557–565. 10.1200/JCO.2015.63.0830 PMC4872028 26644527

