# Supplementary material for: Using electronic patient reported outcomes (ePROs) to improve supportive care in ambulatory oncology care: a CFIR-guided pre-implementation formative evaluation
Source: J Patient Rep Outcomes. 2026 Jul 21;10:194. doi: 10.1186/s41687-026-01144-8 (PMC13677648; doi:10.1186/s41687-026-01144-8)
Supplement: Supplementary file 1 [file 41687_2026_1144_MOESM1_ESM.docx]

**SUPPLEMENTAL MATERIALS**

**Supplementary Table S1:** Consolidated Criteria for Reporting Qualitative Research (COREQ) Checklist

| **Item** | **Guide Question/Description** | **Reported (Location)** |
| --- | --- | --- |
| **DOMAIN 1: RESEARCH TEAM AND REFLEXIVITY** |  |  |
| **Personal Characteristics** |  |  |
| 1. Interviewer/facilitator | Which author(s) conducted the interview or focus group? | PhD-trained clinical scientist with qualitative research expertise and trained research staff members (Methods) |
| 2. Credentials | What were the researcher's credentials? | PhD, MSN, RN and MSN, RN |
| 3. Occupation | What was their occupation at the time of the study? | Clinical researcher, research coordinator, nurse scientist |
| 4. Gender | Was the researcher male or female? | Female |
| 5. Experience and training | What experience or training did the researcher have? | Qualitative research training; prior experience conducting semi-structured interviews and analyzing qualitative data (Methods) |
| **Relationship with Participants** |  |  |
| 6. Relationship established | Was a relationship established prior to study commencement? | No prior relationship with participants |
| 7. Participant knowledge of the interviewer | What did the participants know about the researcher? | Participants were informed of interviewers’ names and their role as nurses/researchers affiliated with the institution; teleconference format allowed observation of interviewer |
| 8. Interviewer characteristics | What characteristics were reported about the interviewer/facilitator? | Professional background. Reflexive discussions acknowledged healthcare provider perspective; some team members with personal experience as family caregivers in cancer contexts |
| **DOMAIN 2: STUDY DESIGN** |  |  |
| **Theoretical Framework** |  |  |
| 9. Methodological orientation and theory | What methodological orientation was stated to underpin the study? | Directed content analysis guided by CFIR; rapid qualitative analysis with matrix summarization (Methods: Data Analysis) |
| **Participant Selection** |  |  |
| 10. Sampling | How were participants selected? | Purposeful and convenience sampling (Methods: Participants and Recruitment) |
| 11. Method of approach | How were participants approached? | Clinical staff via email invitation; patients via email or letter (Methods: Participants and Recruitment) |
| 12. Sample size | How many participants were in the study? | n=46 (20 clinical staff, 26 patients) (Results: Participant Characteristics) |
| 13. Non-participation | How many people refused to participate or dropped out? | No participants dropped out or withdrew after enrollment. |
| **Setting** |  |  |
| 14. Setting of data collection | Where was the data collected? | Teleconference (Zoom) or telephone per participant preference (Methods: Data Collection) |
| 15. Presence of non-participants | Was anyone else present besides the participants and researchers? | For one interview, a family member was present at the request of the participant. |
| 16. Description of sample | What are the important characteristics of the sample? | Tables 1 and 2 (Results: Participant Characteristics) |
| **Data Collection** |  |  |
| 17. Interview guide | Were questions, prompts, guides provided by the authors? | CFIR-guided semi-structured interview questions and coding framework (Methods: Data Collection; Supplementary Table) |
| 18. Repeat interviews | Were repeat interviews carried out? | No |
| 19. Audio/visual recording | Did the research use audio or visual recording to collect the data? | Audio-recorded with participant consent (Methods: Data Collection) |
| 20. Field notes | Were field notes made during and/or after the interview? | Field notes documented with interviewer observations. |
| 21. Duration | What was the duration of the interviews? | Mean 53 minutes (range 24–106) (Methods: Data Collection) |
| 22. Data saturation | Was data saturation discussed? | Purposeful sampling guided by CFIR constructs and project timeline; sampling continued until sufficient information obtained to guide design decisions (Methods) |
| 23. Transcripts returned | Were transcripts returned to participants for comment and/or correction? | Offered as option; none requested. |
| **DOMAIN 3: ANALYSIS AND FINDINGS** |  |  |
| **Data Analysis** |  |  |
| 24. Number of data coders | How many data coders coded the data? | Minimum of two team members independently coded each transcript (Methods: Data Analysis) |
| 25. Description of the coding tree | Did authors provide a description of the coding tree? | CFIR-based codebook with constructs operationalized to ePRO implementation context (Methods: Data Analysis; Supplementary Table) |
| 26. Derivation of themes | Were themes identified in advance or derived from the data? | Deductive approach using CFIR constructs; themes identified within pre-specified construct categories (Methods: Data Analysis) |
| 27. Software | What software, if applicable, was used to manage the data? | Atlas.ti for qualitative data (Methods: Data Analysis) |
| 28. Participant checking | Did participants provide feedback on the findings? | No formal member checking; iterative feedback cycles with clinical staff during implementation planning (Supplementary Materials) |
| **Reporting** |  |  |
| 29. Quotations presented | Were participant quotations presented to illustrate the themes/findings? | Quotes presented in Table 3 (Results) |
| 30. Data and findings consistent | Was there consistency between the data presented and the findings? | Quotes directly support CFIR construct summaries |
| 31. Clarity of major themes | Were major themes clearly presented in the findings? | Themes organized by CFIR domain and construct (Results: Qualitative Findings) |
| 32. Clarity of minor themes | Is there a description of diverse cases or discussion of minor themes? | Areas of convergence and divergence between user groups described (Results: Integration of Findings) |

**Supplementary Table S2:** Standards for Reporting Implementation Studies (StaRI) Checklist: Elements Applicable to Pre-Implementation Formative Evaluation

| **Item** | **StaRI Element** | **Reported (Location)** |
| --- | --- | --- |
| **TITLE** |  |  |
| 1. Identification as implementation study | Identification as an implementation study and description of methodology | Title identifies as pre-implementation formative evaluation; CFIR methodology stated (Title, Abstract) |
| **ABSTRACT** |  |  |
| 2. Structured summary | Identification of implementation research question and methods | Abstract describes CFIR-guided evaluation, methods, and pre-implementation outcomes (Abstract) |
| **INTRODUCTION** |  |  |
| 3. Description of problem | Description of the evidence-practice gap | Gap between ePRO evidence and routine implementation; lack of CDS integration (Introduction) |
| 4. Rationale | Rationale for intervention and implementation strategy | ePRO+CDS system rationale; CFIR for pre-implementation evaluation (Introduction) |
| 5. Aims | Specific objectives, including implementation objectives | Inform design requirements and implementation strategies for ePRO+CDS system (Introduction) |
| **METHODS** |  |  |
| 6. Study design | Design type and rationale | Pre-implementation formative evaluation using mixed methods; CFIR-guided (Methods: Study Design) |
| 7. Context | Characteristics of setting and context factors | Single academic medical center; NCI-designated cancer center; HNC as use case; current practices described (Methods: Setting and Context) |
| 8. Intervention | Description of the intervention | Modular ePRO+CDS system; EHR-integrated; CDS for supportive care referrals (Methods: Prototype Development) |
| 9. Implementation strategies | Description of strategies to support implementation | Pre-implementation phase: Strategies identified through formative evaluation (Table 5) |
| 10. Outcomes | Primary and secondary outcomes | Pre-implementation outcomes: Acceptability (AIM), Appropriateness (IAM), Feasibility (FIM), Usability (SUS) (Methods) |
| 11. Sample size | Rationale for sample size | Pragmatic considerations; recommendations for framework-guided qualitative analysis (Methods) |
| 12. Recruitment and sampling | Recruitment and sampling methods | Purposeful sampling; clinical staff via email; patients via email or letter (Methods) |
| 13. Data collection | Data collection methods and instruments | CFIR-guided semi-structured interviews with prototype demonstration; pre-implementation metrics (Methods) |
| 14. Analysis | Methods of analysis | CFIR-guided directed content analysis with matrix analysis; descriptive statistics (Methods) |
| **RESULTS** |  |  |
| 15. Reach/recruitment | Flow of participants | n=46 participants (Results) |
| 16. Context | Contextual factors affecting implementation | CFIR domains and constructs (Results) |
| 17. Main findings | Implementation and intervention outcomes | Qualitative themes by CFIR construct; quantitative metrics (AIM, IAM, FIM, SUS) (Results) |
| 18. Adaptations | Adaptations to intervention or strategies | Not applicable (pre-implementation phase) |
| 19. Fidelity | Fidelity to implementation strategies | Not applicable (pre-implementation phase) |
| 20. Costs | Resource use and costs | Not applicable (pre-implementation phase) |
| **DISCUSSION** |  |  |
| 21. Summary of findings | Summary of findings and relationship to objectives | Key findings summarized (Discussion) |
| 22. Interpretation | Interpretation of findings in relation to implementation | Findings interpreted in context of prior ePRO implementation literature (Discussion) |
| 23. Generalizability | Discussion of generalizability to other contexts | Single-site limitation acknowledged; adaptation potential discussed (Discussion) |
| 24. Limitations | Limitations and potential biases | Single academic center; patient sample characteristics; HNC-specific focus (Discussion) |
| 25. Implications | Implications for future implementation research | Recommendations for implementation (Discussion) |
| **OTHER** |  |  |
| 26. Funding | Sources of funding and role of funders | Funding information provided |
| 27. Conflicts of interest | Conflicts of interest | COI information provided |

**Supplementary Table S3.** CFIR-Guided Interview Questions and Coding Framework

| **INTERVIEW QUESTION (#)** | **CFIR DOMAIN** | **CFIR CONSTRUCT** | **PROJECT DOMAIN** |
| --- | --- | --- | --- |
| Do you think this type of symptom screening tool could be effective in your clinical practice? Why or why not? | Inner Setting | Compatibility | Tool Efficiency / Fit |
| Do you see a need for this type of tool in your practice with HNC patients? Why or why not? | Inner Setting | Tension for Change | Need for Tool |
| How do you typically assess symptoms in your encounters with HNC patients? | Inner Setting | Usual Practice | Current Symptom Management Practice |
| Are you satisfied with your approach? If so/not, why? | Inner Setting | Tension for Change | Level of Satisfaction with Current Approach |
| How does this fit into existing processes and workflows in your practice? | Inner Setting | Compatibility | Tool Compatibility with Current Clinical Practice |
| How does this fit into existing processes and routines in your daily life? | Inner Setting | Compatibility | Tool Compatibility with Current Clinical Practice |
| What are potential issues or obstacles you can imagine for providers? | Inner Setting | Compatibility | Potential Provider Obstacles To Tool Usage |
| What are potential issues or obstacles you can imagine for patients? | Inner Setting | Compatibility | Potential Patient Obstacles to Tool Usage |
| How do you feel about this tool being used in your practice setting? | Inner Setting | Compatibility | Provider Thoughts on Implementation |
| What suggestions do you have about implementing this tool successfully in your practice? | Implementation Process | Planning | Tool Implementation Recommendations |
| What resources would be necessary for successful implementation? | Inner Setting | Available Resources | Resources for Tool Implementation |
| If you were designing a brief symptom screening tool for HNC patients, what essential symptoms should be included? | Individuals | Innovation Recipients | Patient Symptoms for Inclusion in Tool |
| With these added items, how well would this type of composite tool meet your needs and the needs of HNC patients in your clinic? In what ways? | Individuals | Innovation Recipients | Provider Interest Level in CDS |
| How does this tool compare to other tools you are familiar with? What advantages/disadvantages does this new tool have? | Innovation | Relative Advantage | Provider Needs for CDS Implementation |
| If this type of symptom screening tool was implemented in your clinic, would you be interested in/ prefer to have accompanying clinical decision support to help respond to the patients’ answers on the symptom questionnaire? Why or why not? | Inner Setting  Innovation | Tension for Change  Relative Advantage | Provider Needs for CDS Implementation |
| What would be important to you when using decision support in this context? What are possible concerns when considering decision support for a symptom screening tool? | Individuals | Innovation Deliverers | Provider Needs for CDS Implementation |
| When you think about the symptoms/concerns that are included in the composite screening tool, do you feel more confident/comfortable addressing certain symptoms versus others? | Individuals | Innovation Deliverers | Straightforward symptoms to address |
| Which symptoms do find most challenging to address with your patients? | Individuals | Innovation Deliverers | Challenging Symptoms to Address |
| What resources and referrals do you commonly use to address these issues in your practice? | Individuals  Inner Setting | Innovation Deliverers  Usual Practice | Common Referrals Placed |
